# Adverse event reporting practices in drug-resistant tuberculosis facilities across South Africa

**DOI:** 10.4102/sajid.v38i1.564

**Published:** 2023-12-19

**Authors:** Razia Gaida, Adlai S. Davids, Ronel Sewpaul

**Affiliations:** 1 Department of Public Health, Societies and Belonging, Human Sciences Research Council, Pretoria, South Africa; 2 Centre for Community Technologies, Faculty of Engineering, Built Environment and Technology, Nelson Mandela University, Gqeberha, South Africa; 3 Faculty of Health Sciences, Nelson Mandela University, Gqeberha, South Africa; 4 Department of Public Health, Societies and Belonging, Human Sciences Research Council, Cape Town, South Africa

**Keywords:** pharmacovigilance, tuberculosis, adverse events, drug resistant, reporting

## Abstract

**Background:**

The reporting of adverse drug reactions associated with drug-resistant tuberculosis (DR-TB) medication is important for pharmacovigilance, especially in high-burden countries such as South Africa. With DR-TB treatment being so dynamic, it is important to understand adverse event reporting practices at specialised facilities.

**Objectives:**

The study aimed to understand the adverse drug reaction (ADR) reporting practices at DR-TB treatment facilities in South Africa.

**Method:**

Interviews were conducted with healthcare workers at specialised DR-TB facilities. This was to collect data on demographics, pharmacovigilance training, and determine attitudes and practices towards reporting adverse events. A checklist was developed to review the most recent adverse event forms captured at the facility.

**Results:**

Most participants did not have adverse event reporting training since their initial training but were confident that they could complete a form themselves. Most participants could correctly identify the major adverse events associated with DR-TB medication, but some deemed non-adverse events as plausible. Adverse event report forms were not standardised with most participants deeming further training and regular feedback as reasons to report ADRs.

**Conclusion:**

Standardisation of adverse event report forms used and the establishment of regular reporting will increase adverse event reporting at DR-TB facilities. Continuous training, empowerment and expansion of staff categories eligible to report adverse events will enhance and sustain such practice.

**Contribution:**

The study highlights challenges faced by healthcare professionals in reporting adverse events.

## Background

The incidences of adverse drug reactions (ADRs) of medication used for the treatment of tuberculosis (TB), including various forms of drug-resistant tuberculosis (DR-TB), are well documented. Timely and correct reporting of TB medication ADRs is key to identifying and addressing adverse effects of these medicines. Thus, the need for pharmacovigilance, defined as ‘the science and activities relating to the detection, assessment, understanding and prevention of adverse effects and all other problems related to medicines’,^1^ is very relevant in high-burden TB countries. This scope of pharmacovigilance has expanded, from simply adverse reactions or events, to the World Health Organization’s definition of:

[*V*]arious aspects of medication safety including medication errors, counterfeit or substandard medication, lack of efficacy of medication, the misuse or abuse of medication and the interaction between medications and include the surveillance of herbal products, other traditional and complimentary medication, biologicals, vaccines, blood products and medical devices.^2^

The World Health Organization (WHO) reported that South Africa had an estimated 21 000 cases of multidrug-resistant and rifampicin-resistant TB (MDR or RR-TB) in 2021^3^ with a 78% treatment success rate for the 2019 cohort. Adverse drug reactions are a major challenge facing South Africa’s health system resulting in unnecessary complications in patients and sometimes death.^4,5,6^ Adverse drug reactions contribute to lowered quality-of-life scores in patients with DR-TB, especially during the early stages of treatment and can ultimately impact treatment outcome.^7,8,9^ The need for the successful treatment of MDR/RR-TB is a significant objective for the National Strategic Plan (NSP) for HIV, TB and sexually transmitted infections (STIs), with the additional objective of strengthening ADR reporting and the use of pharmacovigilance reports towards improving patient safety.^10^

The treatment of DR-TB is constantly changing to accommodate new evidence, thus increasing the need for functional active pharmacovigilance programmes at hospital and primary healthcare level due to the decentralisation of DR-TB services. The newly adopted bedaquiline, pretomanid, linezolid and levofloxacin (BPaL-L) requires intensive monitoring of cardiac function, peripheral neuropathy and haemoglobin to ensure early detection and management of potentially serious ADRs.^11^ Adverse drug reactions related to DR-TB create the risk that the patient may stop treatment, particularly in the early stages, leading to ongoing transmission with possible new drug resistance, thus exacerbating the ongoing public health crisis of TB. The aim of the study was to understand the ADR reporting practices at DR-TB treatment facilities in South Africa.

## Methods

This descriptive and cross-sectional quantitative study was undertaken in collaboration with the South African Department of Health. Data were collected from healthcare workers at specialised DR-TB facilities in South Africa, between June and November 2019.

The South African Health Products Regulatory Authority (SAHPRA) provides a guideline on ADR reporting for healthcare professionals.^12^ The ADR monitoring system was established in South Africa in 1987 and is coordinated by the National Adverse Event Drug Monitoring Centre (NAMDEC). The unit collaborates with other programmatic units in the National Department of Health including the Extended Programme for Immunisation Unit and the Department of Health Pharmacovigilance Centre for Public Health Programmes. The Department of Health has a standard ADR report form, but some facilities use tailored forms for specific diseases such as TB for ease of completion. It is also possible to submit forms digitally using the Essential Medicines List application.

The National Pharmacovigilance Centre (NPC) is responsible for the receipt and monitoring of the TB and HIV ADR reports. The National TB Programme requires ADRs of Grade 3 and higher to be reported to the NPC. Grade 1 and 2 ADRs are to be noted in the patient’s medical record.

### Study population

Ten specialised public sector DR-TB facilities were purposively selected within the nine provinces of South Africa. Frontline healthcare workers such as doctors, nurses and pharmacists were included, as well as managerial and administrative staff such as matrons, facility managers, quality assurance managers and operational managers.

### Data collection and instruments

Two data collection instruments were developed. Firstly, a structured questionnaire aimed at healthcare professionals, based on those used in previous studies (Bharadwaj et al.^13^ and Bhagavathula et al.^14^) and guidelines from the SAHPRA.^12^ The questionnaire contained sections on demographics, pharmacovigilance training and ADR reporting, attitudes towards and knowledge of pharmacovigilance and current practices for ADR reporting. Completion of this instrument was done through in-person interviews by trained study staff.

Secondly, a checklist was developed to review ADR reports completed between January and June 2019 at each facility. The items on this checklist were based on the required information stipulated by SAHPRA and NPC of the Department of Health to check for completeness and adherence to requirements for a complete form. The objective was that between 2% and 5% of the available forms would be analysed, but due to the small numbers of completed forms, all forms at each facility were analysed. Information required on ADR report forms included facility name and district, patient details, DR-TB registration number and hospital number together with basic information such as age, height, weight and sex, as well as any reported allergies and current pregnancy. Comorbidities and medication and HIV status were required with viral load, CD4 count and antiretroviral treatment regimen, if applicable. A list of medication prescribed for DR-TB treatment was needed with details such as dose, frequency and mode of administration. An explanation of the ADR, its management and subsequent outcome was required. Full versions of the data collection tools are available in the larger report.^15^

### Analysis

The questionnaires and checklists were coded and captured in spreadsheets. Data were then processed and analysed using Stata 14.0 (StataCorp. 2015. College Station, TX). The primary outcome variables included understanding of the concept of pharmacovigilance, actual knowledge of ADRs associated with medication used to treat DR-TB at the time of the study, reporting practices in the facility and perceptions of the potential impact of reporting ADRs both within the facility and externally. Mediator and moderator variables included the category of health professional and their number of years of experience, both in their profession and at the facility. Basic frequencies were calculated and cross-tabulations of interest were performed. Differences between subgroups tested using chi-square tests and ANOVA were needed.

### Ethical considerations

Research ethics for the study was obtained from the research ethics committee (REC) of the Human Sciences Research Council (HSRC) (Reference: 6/22/08/18). Ethical clearance and permission to access the participating facilities were granted by the provincial and district Departments of Health of each province through the National Health Research Ethics Council (NHREC). Potential participants were provided with relevant information about the study and allowed to ask questions for clarity before providing written consent.

## Results

### Interviews with healthcare professionals

A total of 164 healthcare workers were interviewed. The demographics are summarised in Table 1. Nurses made up the majority of the study population (59.8%), followed by doctors (19.5%) and pharmacists (10.4%). Other categories included matron (6.7%), facility manager (0.6%), quality assurance manager (2.4%) and occupational health nurse (0.6%). Most (43.3%) participants had been working at the DR-TB facility for between 1 and 5 years, and more than half (53.0%) had over 10 years of total work experience in their respective professions.

**TABLE 1:** Demographics (*n* = 164).

Variables	*n*	%
Males	26.0	15.9
Average age (range)	43.6	23–76
Nurses	98.0	59.8
Doctors	32.0	19.5
Pharmacists	17.0	10.4
Other†	17.0	10.4
Urban setting	162.0	98.8
**Years at the facility**		
Less than 1 year	31.0	18.9
1–5 years	71.0	43.3
6–10 years	37.0	22.6
Longer than 10 years	25.0	15.2
**Total years of experience**		
Less than 1 year	9.0	5.5
1–5 years	39.0	23.8
6–10 years	29.0	17.7
Longer than 10 years	87.0	53.0
**Pharmacovigilance training**		
During tertiary education	102.0	62.2
Received adverse effect training in the last 12 months	73.0	44.5
Received training on medication used to treat drug-resistant tuberculosis	108.0	65.9

† , Includes matrons, facility manager, quality assurance manager and occupational health nurse.

**FIGURE 1:**
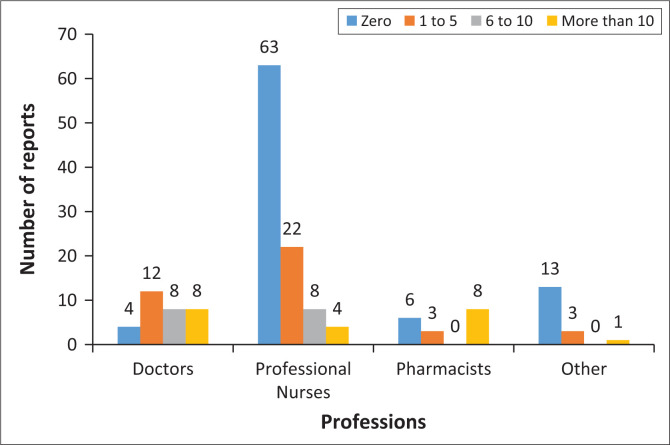
Number of adverse event report forms completed in the last6 months by profession.

While most participants (62.2%) were introduced to pharmacovigilance during their tertiary education, only 39.0% conveyed having received any training in the field thereafter. There was no noticeable difference observed between various professions regarding training in pharmacovigilance. Although the vast majority of participants (99.4%) concurred that reporting of ADRs was an important requirement and that they had a personal obligation to do so (98.8%), a significant portion (52.4%) had not completed an ADR report form during the 6 months preceding the interview. Notably, nurses displayed a lower rate of ADR form completion compared to doctors, while pharmacists fell in between. Figure 1 summarises the number of ADR forms that were completed by profession.

Most participants (72.2%) stated that they could complete an adverse event report form without assistance, and while the forms used varied between facilities, the Department of Health Standard Adverse Event Report Form was most commonly used (59.1%). However, there were intra-facility discrepancies at six facilities for which form was used, with some staff reporting use of the standard Department of Health form while others reported that a TB-specific form was available. Only 14.6% of participants reported completing a TB-specific form. Drug-resistant tuberculosis–specific forms are prepopulated with the complete list of DR-TB medications together with a list of potential ADRs for ease of completion, while the standard form is blank.

There were different views as to which professional category had the primary responsibility to complete adverse event report forms. The majority of the participants reported that this responsibility lay with nurses (95.1%) and doctors (90.9%), rather than with pharmacists (70.1%) or other healthcare professionals (41.4%). Participants were of the view that documenting an ADR within their establishment was a relatively uncomplicated process; yet less than half (48.8%) of the participants believed that their submissions had an impact on the clinical practices at their respective institution. All doctors felt that they could complete forms independently, while less than two-thirds (62.8%) of nurses felt likewise. More than half of nurses (54.9%) indicated that they would report the ADR to someone else and whom they would expect to report. Just 75% of pharmacists felt confident that they could complete a form independently. These values did not differ even with more years of experience. Once the ADR report forms were completed, most participants (41.5%) stated that they handed it to an appointed person in the facility, often the pharmacist, while others (34.8%), particularly doctors (40.6%) and nurses (35.7%), reported inserting the document into the patient’s medical folder where it remained.

Just over three quarters (75.6%) of the study population believed that there was a risk to patient safety because the medication was used to treat DR-TB, but fewer nurses felt that there was a risk compared to other professions. Majority of the participants (71.3%) reported that they were knowledgeable enough about DR-TB medication to identify an ADR. However, nurses (69.4%) were not as confident as doctors (96.9%) and pharmacists (82.4%) in this respect. When participants were asked to identify common ADRs associated with DR-TB medication, all demonstrated good knowledge. The ADRs correctly identified were gastrointestinal disturbances (99.4%), peripheral neuropathy (99.4%), hepatic abnormalities (98.8%), skin reactions (98.2%), ocular toxicity (90.2%), psychosis (97.0%), electrolyte imbalance (93.3%), anaemia (93.3%), musculoskeletal pain (90.9%) and renal abnormalities (98.2%). Adverse drug reactions that participants were less sure of included gynaecomastia (47.0%) and hyperuricaemia (54.3%). The list also included diabetes (31.1%) and bronchospasm (53.0%) which are not associated with DR-TB medication. Significantly fewer participants reported diabetes (31.5%) as an adverse effect of drug-resistant TB medication compared to all the other known effects/events (*p* < 0.05). When comparing the identification of bronchospasm to other ADRs, significantly fewer participants reported bronchospasm (53%) as an adverse effect compared to all the other known events (*p* < 0.05).

In terms of the sources for identifying ADRs, most participants (79.9%) indicated that their patients reported ADRs to them, others were told by a colleague (75.6%) or detected it themselves while physically examining the patient (71.3%). Doctors and professional nurses detected ADRs through these methods while pharmacists relied more on colleagues’ reports of ADRs and examination of the patient’s medical record.

**FIGURE 2:**
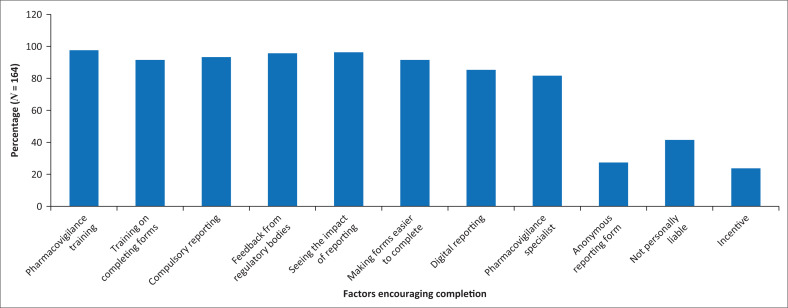
Factors that would encourage completing adverse drug reaction report forms among healthcare professionals.

The ADR report form requires details on how the ADR was managed and the outcome. Participants said that clinical guidelines, consultation with other colleagues and using their own clinical judgement were how they decided how best to manage the ADR. Doctors agreed that they employed all three methods, while nurses and pharmacists preferred consulting clinical guidelines for advice, as some aspects of patient management may not fall within their scopes of practice. Almost half of the participants were unsure if their completed ADR report forms were shared with a regulatory body, and 86.2% of participants who reported an ADR stated that they had not received feedback from a regulatory body. However, more than half (52.2%) of the doctors seemed sure that their reports were shared with a regulatory body. Figure 2 outlines the factors that health professionals said would motivate them to complete ADR report forms.

Training on the relevance of pharmacovigilance (97.6%), seeing the effects of reporting on how patients and ADRs are subsequently managed (96.3%) and receiving feedback from regulatory bodies (95.7%) were the top three factors that would encourage reporting of ADRs. Not being held personally liable (41.5%), the use of anonymous reporting forms (27.4%) and incentives (23.7%) were not likely to encourage completion of ADR forms.

### Review of adverse drug reaction report forms

Seventy-eight reports were analysed to review the ADR reports completed across the 10 DR-TB facilities and are summarised in Table 2.

**TABLE 2:** Number of completed adverse event report forms per site.

Site	Number of completed adverse event report forms
Site 1	14
Site 2	10
Site 3	10
Site 4	7
Site 5	10
Site 6	5
Site 7	4
Site 8	6
Site 9	2
Site 10	10
**Total**	**78**

There are four absolute minimum requirements for an ADR report form to be considered valid. These are a patient identifier such as age, gender, identification number, the identity of the reporter, the suspected medication and the ADR.^12^ A total of 65 (83.3%) reports fulfilled these minimum requirements, but for the purposes of causality assessment, this is insufficient. All facilities, with the exception of one, used the standard ADR report form. One facility used the DR-TB specific form with prepopulated DR-TB medication names and standard doses.

Analysis of the forms showed that identifying data, such as hospital number, was incomplete on 80.7% (*n* = 68) of forms and the DR-TB registration number was incomplete on 35.9% (*n* = 28) of forms. Other information that was often missing were the HIV status (53.8%; *n* = 42), initiation on ART (71.8%; *n* = 56), specific ART regimen (75.6%; *n* = 59), viral load (82.1%; *n* = 64) and other concomitant conditions (89.7%; *n* = 70), all which have a bearing on the reporting of adverse events associated with DR-TB.

## Discussion

The current study aimed to explore the ADR reporting practices at DR-TB facilities throughout South Africa. The majority of the respondents were professional nurses, who are the majority of public sector healthcare workers in South Africa.

Adverse drug reactions of DR-TB medication must first be identified in order to report. This may be challenging given the ADR overlap with antiretrovirals. Health professionals with direct contact with the patient – such as doctors and nurses – identified ADRs through physical examination and interviewing the patient together with analysis of laboratory results. Pharmacists, who are not always patient-facing, detected ADRs through analysing notes in the medical record. Encouraging a multidisciplinary effort during ward rounds may assist in detecting the ADR earlier, thus mitigating serious outcomes.^16^ Although only serious ADRs need to be reported, healthcare professionals should be aware of their patients’ experience as ADRs can reduce adherence.^17^ It is important to include information about possible ADRs during patient education and counselling sessions at initiation and during treatment,^18^ and encourage patients to report them to their healthcare professional at their next visit for appropriate intervention as a part of practising patient-centred care. This is especially important for those with HIV, as the combination of medications may increase the likelihood of ADRs. Pharmacists should be strongly considered to perform this education as they have an in-depth knowledge of the medication prescribed.

While healthcare professionals believed in the importance of ADR reporting, the actual reporting frequency in this study was low. This may reflect the low frequency of ADRs occurring during the period of interest, but Joubert and Naidoo^19^ found similarly low levels of ADR reporting in a study of pharmacists in the North West province of South Africa. The lack of feedback from regulatory bodies, the perceived lack of impact of the reports and the lack of understanding of which health professionals are able to report ADRs may be reasons for low reporting rates. Terblanche^20^ noted that possible reasons for low reporting rates were that health professionals desired incentives to report and their fear of litigation should the ADR be attributed to their practice. Another study^21^ reported that a lack of training on pharmacovigilance activities and how to report ADRs deterred health professionals from reporting and, although there was belief that reporting was important, translating knowledge into practice was challenging. Additionally, while ADR report forms were completed, whether or not this information was shared with regulatory bodies was unknown to most participants.

The review of the completed ADR reports showed that while most sections were fully completed, details about comorbidities, especially HIV and its treatment, were often not available. Given the high burden of TB/HIV co-infection in South Africa, TB medication and antiretrovirals are regularly prescribed concomitantly. Providing complete information about the patient would assist with signal detection and causality assessments.

A thorough understanding of the need and process, regular training and refresher seminars as well as inclusion in the feedback cycle would be beneficial to encourage reporting among health professionals. Similar results were found by Evans et al.^22^ and Gupta et al.^23^ where a lack of training and feedback were deterrents to reporting. Such training will be especially relevant given the 95-95-95 objective set in the NSP for 2023–2028. The NSP states that interventions to reach that objective include the enhancement of side effect detection and management, the reinforcement of adverse reaction reporting to drugs, and utilisation of pharmacovigilance reports.^24^

## Conclusion

The need for standardisation of ADR report forms and the establishment of a clear and regular reporting feedback loop will increase ADR reporting at DR-TB facilities in South Africa. The continuous training, empowerment and expansion of staff categories which can report such adverse events will improve practice.
